# Supplementary material for: Patterns of Intron Gain and Loss in Fungi
Source: PLoS Biol. 2004 Nov 30;2(12):e422. doi: 10.1371/journal.pbio.0020422 (PMC532390; doi:10.1371/journal.pbio.0020422)
Supplement: Table S1 — Also available at http://genes.mit.edu/NielsenEtAl/. (4.3 MB ZIP). [file pbio.0020422.st001.zip › NielsenEtAl/html/18.html]

AN7254.1.NCU00018.1.MG05193.1.FG05530.1


```
 CLUSTAL W (1.82) Multiple Sequence Alignments - Introns Inserted


Sequence 1: MG05193.1	820 aa
Sequence 2: FG05530.1	821 aa
Sequence 3: NCU00018.1	824 aa
Sequence 4: AN7254.1	827 aa
Alignment Length: 831 aa
Number Identitical Residues: 671 aa
Alignment Score (without introns) 26961


MG05193.1 	-----MSADPSQDPP--KKKVNLA2DVSGADVKE0ESDVATAILKKKKKPNQLM2VADAT
NCU00018.1	-----MAPEPNPDHVEQKKKVNLM2DASGAEHKA0EDDTATAILKKKKKPNQLM2VTDAV
FG05530.1 	-----MAPQPDEHHP--HKKVNLT2DASGAEVKN0EDDVATAILKKKKKPNQLM2VTDAV
AN7254.1  	MCCWGGSRSGTLNLCVNSRRVYVA2DASGAEKKE0ELDTSTAILKKKKKPNSLI2VTDAV
          	 .. . : .   .   . ::* :  *.***: *  * *.:***********.*: *:**.

MG05193.1 	NDDNSIIALSNSTMEALQLFRGDTVLVRGKKRKDTVLIVLADDELDDGSARLNRVVRHNL
NCU00018.1	NDDNSIIALSNNTMEQLQLFRGDTVLVRGKKRKDTVLIVLADDDLDDGSARLNRVVRHNL
FG05530.1 	NDDNSIIALSEATMDQLQLFRGDTVLVRGKKRKDTVLIVLADDELDDGSARINRVVRHNL
AN7254.1  	NDDNSTISLSNNTMDTLGLFRGDTVTVRGKKRKETVLIVLADDDLDDGSARINRVVRHNL
          	***** *:**: **: * ******* *******:*********:*******:********

MG05193.1 	RVKHGDVVTIHPCPDIKY0AKRIAVLPIADTVEGLTGSLFDVFLAPYFREAYRPVRQGDI
NCU00018.1	RVKHGDIITIHPCPDIKY0AKRIAVLPIADTVEGITGSLFDVFLAPYFREAYRPVRQGDL
FG05530.1 	RVKHGDMITIHPCPDIKY0AKRIAVLPIADTVEGITGSLFDVFLAPYFREAYRPVRQGDL
AN7254.1  	RVKHGDIITVHPCPDIKY0AKRIAVLPIADTVEGLTGSLFDVYLAPYFRDGYRPVKQGDL
          	******::*:******** ***************:*******:******:.****:***:

MG05193.1 	FLVRGGMRQVEFKVVEVDPPEYGIVAQDTVIHCEGEPIQRDEEENNLNEVGYDDIGGCRK
NCU00018.1	FIVRGGMRQVEFKVVEVDPPEYGIVAQDTVIHCEGEPIQRDEEENNLNEVGYDDIGGCRK
FG05530.1 	FIVRGGMRQVEFKVVEVDPPEYGIVAQDTVIHCEGEPIQRDEEENNLNEVGYDDIGGCRK
AN7254.1  	FTVRGGMRQVEFKVVEVDPPEFGIVAPDTIIHSEGEPIQREDEENNLNEVGYDDIGGCRK
          	* *******************:**** **:**.*******::******************

MG05193.1 	QMAQIREMVELPLRHPQLFKSIGIKPPRGVLLYGPPGTGKTLMARAVANETGAFFFLING
NCU00018.1	QMAQIREMVELPLRHPQLFKSIGIKPPRGVLLYGPPGTGKTLMARAVANETGAFFFLING
FG05530.1 	QMAQIREMVELPLRHPQLFKSIGVKPPRGVLLFGPPGTGKTLMARAVANETGAFFFLING
AN7254.1  	QMAQIRELVELPLRHPQLFKSIGIKPPRGILMYGPPGTGKTLMARAVANETGAFFFLING
          	*******:***************:*****:*::***************************

MG05193.1 	PEIMSKMAGESESNLRKAFEEAEKNSPAIIFIDEIDSIAPKREKTNGEVERRVVSQLLTL
NCU00018.1	PEIMSKMAGESESNLRKAFEEAEKNSPAIIFIDEIDSIAPKREKTNGEVERRVVSQLLTL
FG05530.1 	PEIMSKMAGESESNLRKAFEEAEKNSPAIIFIDEIDSIAPKREKTNGEVERRVVSQLLTL
AN7254.1  	PEIMSKMAGESESNLRKAFEEAEKNSPAIIFIDEIDSIAPKREKTNGEVERRVVSQLLTL
          	************************************************************

MG05193.1 	MDGMKARSNVVVMAATNRPNSIDPALRRFGRFDREVDIGIPDPTGRLEILQIHTKNMKLG
NCU00018.1	MDGMKARSNVVVMAATNRPNSIDPALRRFGRFDREVDIGIPDPTGRLEILQIHTKNMKLA
FG05530.1 	MDGMKARSNVVVMAATNRPNSIDPALRRFGRFDREVDIGVPDPTGRLEILQIHTKNMKLG
AN7254.1  	MDGMKARSNVVVMAATNRPNSIDPALRRFGRFDREVDIGIPDPTGRLEILSIHTKNMKLG
          	***************************************:**********.********.

MG05193.1 	DDVDLEQIAAETHGYVGSDVAALCSEAAMQQIREKMDLIDLDEDTIDAEVLDSLGVTMEN
NCU00018.1	DDVDLEQIAAETHGYVGSDIAALCSEAAMQQIREKMDLIDLDEDTIDAEVLDSLGVTQEN
FG05530.1 	DDVDLEQIASETHGYVGSDVAALCSEAAMQQIREKMDLIDLDEDTIDAEVLDSLGVTMEN
AN7254.1  	EDVDLETIAAETHGYVGSDLASLCSEAAMQQIREKMDLIDLDEDTIDAEVLDSLGVTMEN
          	:***** **:*********:*:*********************************** **

MG05193.1 	FRFALGVSNPSALREVAVVEVPNVRWEDIGGLDEVKQDLREQVQYPVDHPEKFLKFGLSP
NCU00018.1	FRFALGVSNPSALREVAVVEVPNVRWEDIGGLETVKQELRESVQYPVDHPEKFLKFGLSP
FG05530.1 	FRFALGVSNPSALREVAVVEVPNVRWEDIGGLQEVKQDLKENVQYPVDHPEKYLKFGMSP
AN7254.1  	FRYALGVSNPSALREVAVVEVPNVRWEDIGGLEEVKRELIESVQYPVDHPEKFQKFGLSP
          	**:*****************************: **::* *.**********: ***:**

MG05193.1 	SRGVLFYGPPGTGKTMLAKAVANECAANFISVKGPELLSMWFGESESNIRDIFDKARAAA
NCU00018.1	SRGVLFYGPPGTGKTMLAKAVANECAANFISVKGPELLSMWFGESESNIRDIFDKARAAA
FG05530.1 	SRGVLFFGPPGTGKTMLAKAVANECAANFISVKGPELLSMWFGESESNIRDIFDKARAAA
AN7254.1  	SRGVLFYGPPGTGKTMLAKAVANECAANFISVKGPELLSMWFGESESNIRDIFDKARAAA
          	******:*****************************************************

MG05193.1 	PCIVFLDELDSIAKARGGSVGDAGGASDRVVNQLLTE1MDGMTSKKNVFVIGATNRPEQL
NCU00018.1	PCVVFLDELDSIAKARGGSVGDAGGASDRVVNQLLTE1MDGMTSKKNVFVIGATNRPEQL
FG05530.1 	PCVVFLDELDSIAKARGGSMGDAGGASDRVVNQLLTE~MDGMTSKKNVFVIGATNRPEQL
AN7254.1  	PCVVFLDELDSIAKSRGGSVGDAGGASDRVVNQLLTE1MDGMTSKKNVFVIGATNRPEQL
          	**:***********:****:***************** **********************

MG05193.1 	DPALCRPGRLDSLIYVPLPDELGRLSILKAQLRKTPVSDDVDLQYIANKTHGFSGADLGF
NCU00018.1	DPALCRPGRLDSLIYVPLPDEAGRLGILKAQLRKTPVAADVDLNYIASKTHGFSGADLGF
FG05530.1 	DPALCRPGRLDSLIYVPLPDEPGRLSIIKAQLRKTPIASDIDFGYIASKTHGFSGADIGF
AN7254.1  	DAALVRPGRLDTLVYVPLPDQASREGILKAQLRKTPVASDVDIEFIASKTHGFSGADLGF
          	*.** ******:*:******: .* .*:********:: *:*: :**.*********:**

MG05193.1 	ITQRAVKIAIKESITADINRTKALEAAGEDVPMD--EDAEDPVPELTKRHFEEAMQQARK
NCU00018.1	ITQRAVKIAIKESITADIQRTKEREAAGEDVEME--DEVEDPVPELTKRHFEEAMSMARR
FG05530.1 	ITQRAVKIAIKESIAIDIERQKAREAAGDEMDTD--EDAEDPVPELTKAHFEEAMQMARR
AN7254.1  	VTQRAVKLAIKESISAEIERQKQREAAGEDVKMEDEEEGEDPVPELTRAHFEEAMKTARR
          	:******:******: :*:* *  ****:::  :..:: ********: ******. **:

MG05193.1 	SVSDVEIRRYEAFAQQMKNAGPGAFFKFPE-GEGAPA-ASGGETFNDGGNDDGLYD
NCU00018.1	SVSDVEIRRYEAFSQQMKNAGPGAFFKFPEGGVESSGNGGAGNSFGDAGNDDDLYN
FG05530.1 	SVSDVEIRRYEAFAQQMKNAGPGAFFKFPEAGAEAAG-GDAGNSFGDAGNDDDLYD
AN7254.1  	SVSDVEIRRYEAFAQSLKNSGGSSFFRFPSANEAADS----GNTFGEAGNDDSLYD
          	*************:*.:**:* .:**:**...  : .    *::*.:.****.**:
```
